# The Nurse Role in the Management of ADHD in Children and Adolescent: A Literature Review

**DOI:** 10.3389/fpsyt.2022.676528

**Published:** 2022-02-22

**Authors:** Liv Kleve, Lisa Vårdal, Irene Bircow Elgen

**Affiliations:** ^1^Division of Mental Health, Department of Child and Adolescent Psychiatry, Haukeland University Hospital, Bergen, Norway; ^2^Department of Clinical Medicine, University of Bergen, Bergen, Norway

**Keywords:** ADHD, ADHD management, nurse role, ADHD treatment, inattention

## Abstract

**Objective:**

To review literature regarding existing and recommended roles for nurses in the management of children with ADHD.

**Methods:**

MEDLINE and CINAHL were searched from 2010 to the end of 2019 with the following keywords: ADHD, nurse, child, and inclusion criteria published in an English journal.

**Results:**

Forty-three records were found. However, only five articles scientifically evaluated the nurse role. The nurse role in ADHD management seemed to vary across countries with nurses working independently or as part of a team with delegated responsibility.

**Conclusion:**

The literature review gave information to suggest that nurses can have a significant role in providing a range of medical and non-medical management.

## Introduction

Attention-deficit/hyperactivity disorder (ADHD) is a neurodevelopmental disorder, and for many of the children, this will last until adulthood and is often referred to as a chronic condition (1–3). Symptoms are manifested across several areas of life and may vary between home, school, and leisure activities. Attention-deficit/hyperactivity disorder is also often associated with comorbid conditions (4). Management can be difficult and time consuming, and there is a need to ensure that evidence-based approaches are implemented. The life course perspective in ADHD becomes crucial where detection and intervention in early life have implications for later outcomes (5). Several families also describe their daily living as demanding and ask for additional support with siblings (6).

In 2019, AAP introduced a revised edition of Clinical Practice Guidelines for the diagnosis, evaluation, and treatment of ADHD in children and adolescents (7, 8). The guidelines give several detailed recommendations including comprehensive follow-up programs, in addition to pharmacotherapy and behavioral intervention. The implementation of these extended guidelines challenges child and adolescent mental health services (CAMHS) both in relation to the organization of the service and in how to collaborate across service levels.

Some studies have identified that the shortage of psychiatrists in CAMHS often becomes a bottleneck both for diagnostics and for treatment of ADHD children (4, 9, 10). In response to this, some have suggested that nurses are a resource in the management of ADHD (4). In addition, there is an ongoing discussion whether primary health care should play a more pronounced role in the assessment process (4, 11). Coghill, however, argues that because of the ongoing need to monitor symptoms, impairments, functioning, and comorbidities, the overall responsibility for ADHD should remain within specialist mental health services (4, 11).

Given the above challenges and the fact that ADHD is a major aspect of the work within CAMHS, it is pertinent to explore which of the tasks suggested in the American Psychological Association (AAP) guidelines need to be profession-specific. The aim of this study was to review literature regarding existing and recommended roles for nurses in the assessment and treatment of children with ADHD.

## Methods

### Literature Search

We performed a systematic review of the literature based on the keywords ADHD, nurse, and child. The search strategy was developed in collaboration with a university librarian experienced in systematic literature searches. The searches were conducted in MEDLINE (1946 to February 2020) and CINAHL (1981 to current).

After the first search in MEDLINE and CINAHL, we retrieved 652 articles. Then we removed duplicates and narrowed the search from 2010 to the end of 2019, to 205 articles.

We screened 205 articles, including main and running titles, for nurse role, and ADHD. Of these, 128 records were excluded because of non-relevant topics. For the 77 abstracts, 58 were available and assessed. Reference list for these 58 articles was reviewed, and five more articles were found and included (*n* = 63). Full-text articles were excluded because of other conditions than ADHD, nurse role but not ADHD-specific related, language, or age limitations were 20 years, leaving 43 articles for evaluation.

**Figure 1:**
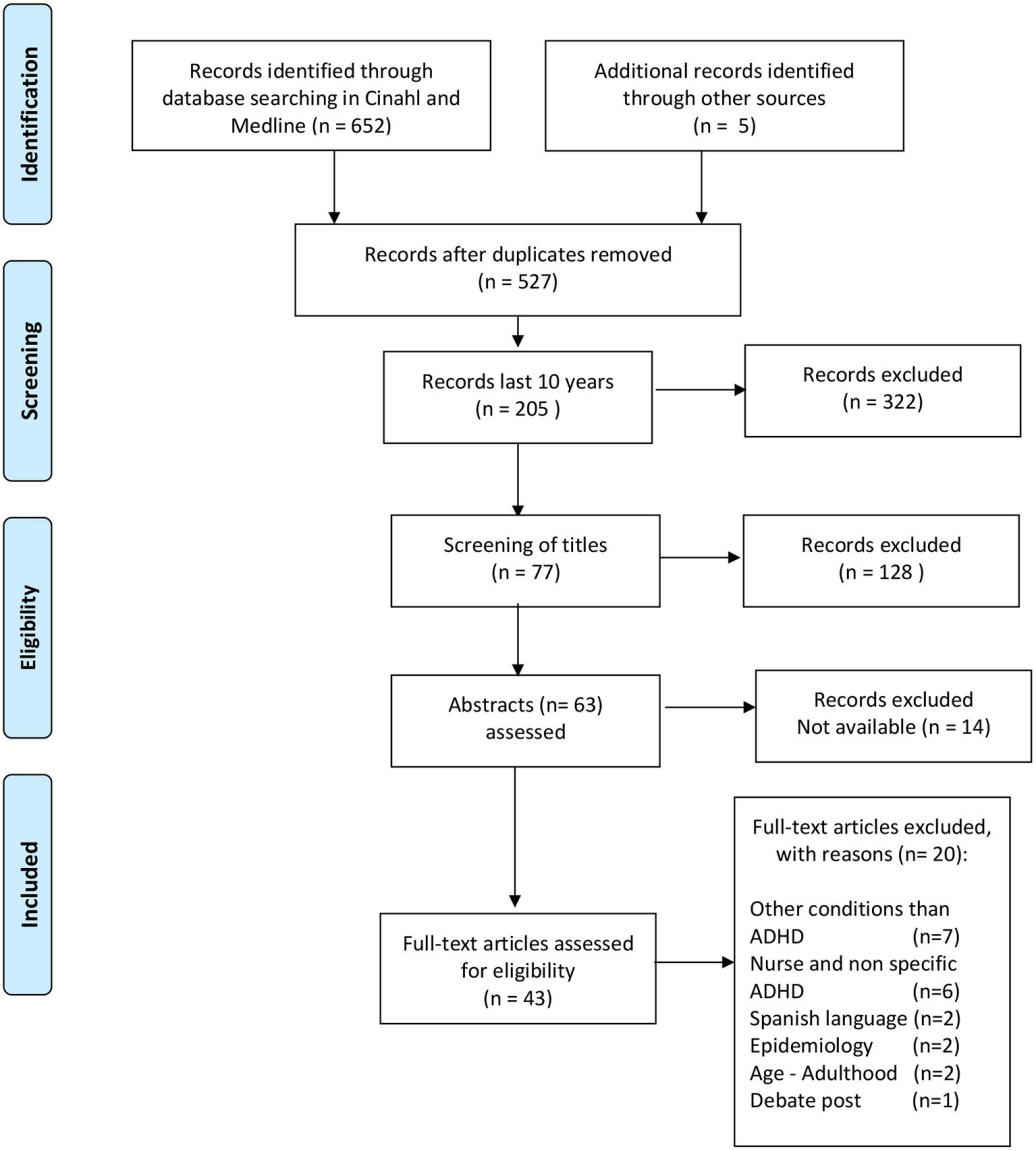
Review of ADHD–Nurse–Child and adolescent.

Results are presented in Figure 1.

### Inclusion Criteria

In order to be included in the review (Figure 1), a study had to (1) be published, or in press, in an English language journal and (2) be available at the University Library of Bergen. Twenty articles identified were excluded from the review because of different reasons, that is, other conditions such as prematurity where ADHD is mentioned (given in Figure 1).

## Results

Results from the literature search are described in the flowchart in Figure 1. Among the 77 identified references, abstract reviewed identified 43 references as eligible for further assessment. In the review process, we identified the following topics:

1. Nurse role description for ADHD assessment, treatment, and management for children and adolescents (Table 1) (1, 2, 4, 6, 10–33).
2. Evaluation of the nurse role (Table 2) (9, 34–37).
3. Articles with a textbook formation presenting knowledge about ADHD and related conditions (Table 3) (3, 38–46).

**Table 1:** Summary of recommendations regarding nurse role in ADHD diagnostic assessment, treatment, and management: a literature review.

• Independent diagnostic assessment and in team context • Independent medical treatment and monitoring ADHD medication and in collaboration with clinicians. • Non-medical treatment: ° Developing coordinated treatment plans involving patient, family, local community, and CAMHS ° Psychoeducation to patients and families ° Behavioral treatment programs ° Care and support to the families, counseling, coping strategies ° Coaching the patient (adolescence) • Educational work in the community i.e., school nurses and teachers • Argued for school nurse to avoid emergency room visit • Argued for nurse as a part of the team • Described nurse led ADHD management • Coghill argued for nurse led treatment of ADHD in specialist health care service • Transitioning from adolescence until adulthood • Argued for nurse led ADHD management instead of physician • Argued for school nurse to take a more active role in ADHD management • Described nurse could help monitoring ADHD medication as well as diagnostic assessment • Argued for nurse to prescribe ADHD medication and organize collaboration with family doctor and CAMHS • SN provide a bridge between CAMHS and school

**Table 2:** Review of studies evaluating nurse role.

• Treatment prescription patterns for nurse and physician with no significant differences • 36 nurses were compared to 34 physician regarding ADHD prescription with no differences • Survey of NP's use of guideline • Nurses can use solution-focused approach when providing care. A mixed design randomized controlled study • Nurse support in medication adherence

**Table 3:** Review of consensus of nurses working with children and adolescents with ADHD.

• What is ADHD? • Comorbidity and ADHD • Medical treatment in ADHD • Side effects of medication • Psycho-education and ADHD • Behavioral therapy and ADHD • Bipolar disorder and ADHD • Sleep apnea • How to identify ADHD school children among school nurses? • Knowledge about school nurse role as an integral part of helping children with ADHD • Auditory processing disorders and ADHD • Neuro-feedback and ADHD

### The Nurse Role in ADHD Management

Articles describing the nurse role identified several aspects such as different nurse titles highlighting that nurses worked in a variety of specialties, that is, school nurse, nurse practitioner, and specialist care nurse. The training requirements for the different nurse specialties, however, varied between countries.

Twenty-eight articles described the content of the nurse role and different tasks (Table 1). These included prescreening and diagnostic assessment for ADHD either as a part of a multidisciplinary team or independently. Further, both medical and non-medical treatments were described and recommended to be delivered by nurses. Several records had more consensus from experts in nurse management of ADHD.

The topic in eight articles related to the organization of ADHD services and the role of nurses in the management of these (Table 1). Themes varied significantly including how to collaborate across service levels (17), running a pilot charity drop in clinic (30), and managing a well-established protocol-driven clinical care pathway based within CAMHS (4). In describing the Dundee ADHD Clinical Care Pathway, Coghill argues for nurse-led continuing care clinics with annual reviews of patients (4, 11). Shared-care agreement as a collaboration between primary and specialist health care is also highlighted (4, 11). Coghill referred to two official audits of a nurse-led care pathway (4, 47, 48), concluding that the pathway was compliant with all the major recommendations of the Scottish Intercollegiate Guideline Network and the National Institute for Clinical Excellence [(49), *Attention Deficit and Hyperkinetic Disorders in Children and Young People. A National Clinical Guideline* (50, 51)].

One thesis explored provider receptiveness to referring patients to a nurse-managed center for the diagnosis and management of ADHD. While most providers expressed willingness to refer to a specialty center for ADHD, a majority of the physician group were negative to refer to a nurse-managed center. It was concluded that the number of potential referrals for establishing this type of center was insufficient (31).

The other thesis (19) assessed five family nurse practitioners and one pediatrician regarding the use and evaluation of a community treatment guideline packet to assist with diagnosing ADHD in children. Overall, providers felt that being more aware of the potential referrals and resources in the community would allow a multimodal approach of care, therefore improving their management of children, aged 4–11 years, with ADHD.

### Evaluation of the Nurse Role

Five articles examined the acceptability/safety of nurse-led ADHD interventions and management (9, 34–37). Summarized in Table 2, only two studies compared nurses' prescription pattern of medication practice with physicians and found no significant differences between the two groups (9, 36). Foreman et al. compared 36 specialist nurses with 34 doctors regarding ADHD care in an ADHD follow-up clinic. Outcomes consisted of predicted diagnostic category from Strengths and Difficulties Questionnaire, patient satisfaction, and side-effect evaluation. Few differences were found, although it was suggested to add structured behavioral screening if nurses were managing the ADHD follow-up. Another survey examined nurses' use of guidelines. For the 89 nurses, approximately 50% of the AAP guideline for assessment and treatment was adhered to, but use of all criteria was less than optimal (34).

One study evaluated a solution-focused approach to increase self-efficacy in ADHD adolescents and supported specialist nurses to carry out this intervention for patients with low self-esteem (35).

Savill et al. described a study of atomoxetine administered by nurse advisers giving support to the patients in order to increase compliance of treatment compared to naturalistic medication. The effect was the result of the support rather than the role of the nurse.

### Professional Opinions

Several articles presented opinions, consensus, or guidelines from experts in nursing, identifying knowledge gaps and suggestions for further training in ADHD management. Suggested training needs for nurses are given in Table 3.

## Discussion

The nurse role in ADHD management seemed to vary, and the requirements for training and certification for specialties within nursing differed between countries. In this review, we found nurses working either independently or as a part of a team with delegated responsibility. With only a few scientific evaluations available, we also included articles describing the nurse role and knowledge gaps. This was done in order to get an overview and gain knowledge in how nursing roles are designed across different countries.

In the present literature review, we found five articles formally evaluating the nurse role with two for medical prescription, one for ADHD follow-up care, one survey of nurses' use of guidelines in diagnostics and treatment, and lastly one evaluating the effect of a nurse-led solution-focused approach. No concerns about safety were identified regarding the prescription and monitoring of ADHD medication. However, more studies are needed.

Nurses' compliance with guidelines was found to be low. In addition, this was not compared with doctors' use of guidelines, making the results difficult to interpret.

Even though no studies were found, several articles described nurses both assessing and diagnosing children for ADHD. Independent assessment by nurses seemed to vary between different regions, and nurses were recommended to undertake specialized training in ADHD.

In addition, two Scottish Healthcare reports included audits of a nurse-led clinical care pathway (Scotland, H.I.). From these reports, it was concluded that long-term follow-up of children and adolescents with ADHD appeared useful, in particular from a user perspective. Regular reviews are identified as crucial, given that ADHD symptoms change during the course of development and during times of change, for instance, during transfer to infant and secondary school. Furthermore, continuity of care has been highlighted as important and recommended to be delivered by nurses. The follow-up of children and adolescents with ADHD is different from other chronic conditions, for example, diabetes mellitus, which can be maintained through simple medical consultations. Impairment associated with ADHD often includes more than core symptoms such as emotional and social impairments in addition to comorbid conditions. Qualitative studies (6, 13) focused on families' need for support and for collaboration across home, school, and primary care.

In this review, we found descriptions of nurses involved with ADHD management in 28 articles. Nurses already carry out many key tasks relating to children and adolescents with ADHD, although the extent of this practice seems to vary between areas and countries. In fact, nurses were recommended to be involved in almost every aspect of ADHD management including assessment and diagnostics, psychosocial support, medical, and non-medical treatment, and in the lead of entire ADHD services. The contribution of nurses was suggested by some authors to involve a caring and supportive quality. Providing support and care may be of particular importance for lifelong chronic conditions (5, 52, 53). Anker found that ADHD outcomes are largely associated with social characteristics and history of depression rather than intellectual functioning or ADHD symptom severity (52).

The reports evaluating a nurse-led care pathway gave favorable reviews of implementation and adherence to clinical guidelines.

Although there is a need for further evaluation and development of the nurse role, there seem to be good indications that nurses are already successfully integrated in teams for children and adolescents with ADHD and that they can provide a life course and family perspective (13) and support in both medical and non-medical treatment. For medical treatment, nurses can have a role in both prescribing medication and the monitoring process.

## Conclusion

This literature review gives information to suggest that nurses can play a major role in providing continuity of care for children and adolescents with ADHD.

## Data Availability Statement

The original contributions presented in the study are included in the article/supplementary material, further inquiries can be directed to the corresponding author/s.

## Author Contributions

LK has participated in preparing the literature search, in reviewing papers, and writing of the manuscript. LV has conducted the literature search. IE has participated in preparing the literature search, reviewing papers, writing of the manuscript, and for the method and result section. All authors contributed to the article and approved the submitted version.

## Conflict of Interest

The authors declare that the research was conducted in the absence of any commercial or financial relationships that could be construed as a potential conflict of interest.

## Publisher's Note

All claims expressed in this article are solely those of the authors and do not necessarily represent those of their affiliated organizations, or those of the publisher, the editors and the reviewers. Any product that may be evaluated in this article, or claim that may be made by its manufacturer, is not guaranteed or endorsed by the publisher.
